# Haitian State Hospital Orthopedic Grand Rounds Series: A Virtual Curriculum to Address Global Surgery Needs

**DOI:** 10.5334/aogh.4304

**Published:** 2023-12-06

**Authors:** Lindsay Hock, Melissa Zahl, Pierre-Marie Woolley, Christina Barau Dejean, Christian A. Pean, Ronald Israelski

**Affiliations:** 1 Touro College of Osteopathic Medicine, Middletown, NY, United States; 2 Department of Orthopaedics, HUP La Paix State University Hospital, Port-au-Prince, Haiti; 3 Department of Orthopaedic Trauma Surgery, Duke University School of Medicine, Durham, NC, United States; 4 Orthopedic Relief Services International (ORSI), United States; 5 Department of Orthopaedic Surgery, Touro College of Osteopathic Medicine, Middletown, NY, United States

**Keywords:** Orthopedic Surgery, Trauma, Global Surgery, Haiti, Surgical Education

## Abstract

**Background:**

Orthopedic Relief Services International (ORSI), in partnership with the Foundation for Orthopedic Trauma and the department of Orthopedic Surgery of La Paix University Hospital in Haiti, has developed a year-round Orthopedic Grand Round series. This series is moderated by Haitian faculty, features presentations by American orthopedic surgeons, and is broadcast to major state hospitals in Haiti for residents and attendings.

**Objective:**

To introduce clinical concepts and increase knowledge in an area that is medically underserved, especially in the field of orthopedics, through lectures that tailor to the educational needs of Haiti.

**Methods:**

Topics for lecture series are requested by Haitian attending orthopedic surgeons and residents in collaboration with American orthopedic surgeons to meet the educational needs of the residents in Haiti. These lectures reflect the case mix typically seen at state hospitals in Haiti and consider the infrastructural capacity of participating centers. Grand rounds are held an average of twice per month for an hour each, encompassing an educational lesson followed by an open forum for questions and case discussion. Feedback is taken from Haitian residents to ensure the sessions are beneficial to their learning.

**Findings and Conclusions:**

To date 95 sessions hosted by 32 lecturers have been completed over Zoom between the US and Haiti. The fourth year of the lecture series is currently ongoing with an expansion of topics. In an underserved medical area such as Haiti, programs that educate local surgeons are crucial to continuing the growth and development of the medical community. Programs like this have the potential to contribute to the educational infrastructure of countries in need, regardless of the specialty. The model of this program can be used to produce similar curricula in various specialties and areas around the world.

## Introduction

For many years Haiti has struggled with some of the worst healthcare conditions in the Western hemisphere [1,2,3]. When the devastating 7.0 magnitude earthquake struck Haiti in 2010, injuring or killing hundreds of thousands of people, this discrepancy in health care was even further highlighted, especially the lack of orthopedic surgeons [1,4]. In 2002, a survey showed that there were only 67 orthopedic surgeons for a population of almost 11 million people, a number that only increased to 127 by 2017 [1,3]. In comparison, the US had 19,001 orthopedic surgeons for a population of 325 million people in 2017 [5]. In addition to a lack of manpower, many other underlying issues further compounded the problem; the lack of proper instruments, implants, and basic operating room equipment created an added challenge to an already difficult time [1]. Some other stressors to a productive and efficient operating room include a lack of basic utilities (electric/clean water) and human resources (i.e. sterilization, product management/inventory personnel) [6,7].

Immediately following the earthquake, hundreds of organizations responded to aid Haiti with orthopedic, trauma, and humanitarian support [8,9]. Although this response was crucial at that time, it did not address the long-standing lack of education, materials, and resources that Haiti had been struggling with [10,11,12].

In 2010, Orthopedic Relief Services International (ORSI), a 501(c)(3) not-for-profit, was founded to address these challenges, in the form of infrastructural, clinical, and educational aid to the State Hospital System in Haiti. To address gaps in education specifically, an Orthopedic Grand Rounds series has been developed that is broadcast from the US to the major state hospitals in Haiti. The lecture series, initiated in 2020, features prominent US orthopedists who are experts in their fields. These lectures cover various orthopedic topics, ranging from specific needs of Haitians to new advancements in the field of orthopedics.

## Methods

The planning process for grand rounds lectures each year begins with Haitian orthopedic surgeons recommending topics of interest to attending orthopedic surgeons at ORSI. With these ideas in mind, US surgeons are recruited to prepare presentations on specific topics. A schedule is prepared by ORSI with speaker names and presentation titles before being finalized in accordance with Haitian program coordinators. This schedule is then distributed by email to all participating Haitian hospitals and orthopedic residency programs.

**Figure 1:**
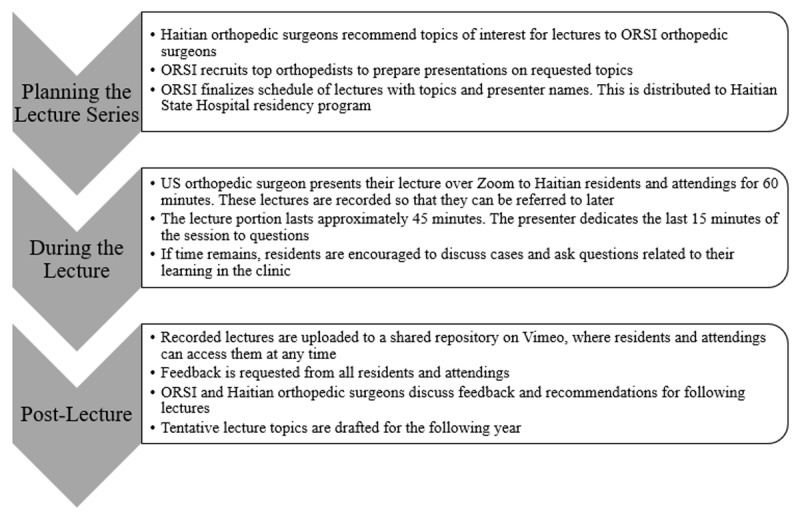
Workflow for Virtual Grand Rounds: Planning, Lecture Design, and Follow-up Post-Lecture.

Grand round sessions are held an average of twice per month and are moderated through the educational program director at La Paix Hospital. Lectures are given in English and are all translated into French in real time. Each session is one hour, consisting of a 45-minute lecture presentation followed by a 15-minute open discussion where residents are encouraged to ask questions (Figure 1). Often, the residents use this time to discuss other orthopedic topics of interest that don’t necessarily pertain directly to the lecture but instead address orthopedic questions they have found within their clinical practice, including case discussions.

Each lecture, including the question and case discussion session, is recorded on the Zoom platform, and uploaded onto the Vimeo online database. The website links are readily available to all Haitian residents, allowing them to access the archived videos at any time.

Feedback is requested on a quarterly basis in the form of an email survey that is distributed to Haitian residents and attendings. Using the results of this survey, the team at ORSI meets with Haitian orthopedic surgeons and program coordinators to discuss feedback and recommend topics for the following year. Tentative lecture topics are drafted for the upcoming year and the process is started again.

## Results

**Figure 2:**
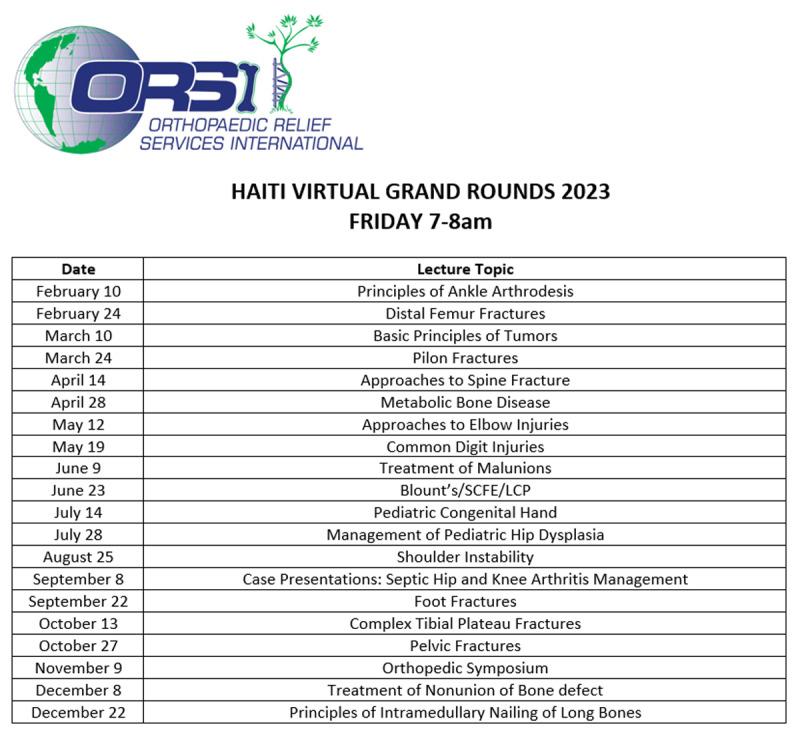
Virtual Grand Rounds schedule for 2023 year, highlighting lecture topics and dates.

Since the initiation of the program in 2020, 95 lectures have been virtually broadcast to Haitian State Hospital residency programs. These topics have ranged from basic orthopedic principles to metabolic and congenital diseases, with each lecture being taught by orthopedic experts from across the US. The cohort of lecturers consists mainly of orthopedists who lecture at least one to two times per year to create a consistent curriculum and is further supplemented with guest lecturers to address newly requested topics. These lectures are continually followed by both ORSI and Haitian program coordinators to ensure consistent quality regardless of who is lecturing. To date, 32 orthopedic experts in their respective fields have prepared and given lectures within this series. Many of these speakers have been a part of the series for years, giving at least one lecture per year. The current fourth year of the series consists of 20 unique grand round lectures, hosted by 10 returning orthopedic experts as well as 10 new guest preceptors (Figure 2).

Feedback, including questions regarding usefulness and practical applications of topics taught, is vetted and has successfully been used to optimize lecture topics for upcoming sessions; presentations have been adapted over time to address the Haitian clinical needs and capabilities more appropriately. While the case mix is skewed towards trauma, coordinators have been careful to include other relevant topics to represent typical orthopedic maladies in Haiti per the request of multiple Haitian residents and attendings. For example, with recent spikes in food insecurity due to civil unrest, starvation and malnutrition have become more prevalent and therefore lectures on metabolic bone disease, seen in malnutrition states, such as rickets, osteomalacia, and scurvy have become more relevant.

Survey results show that the majority of residents find these lectures to be valuable to their education and provide them with a solid foundation in orthopedics. They report that these formal didactic sessions give them access to lectures they wouldn’t see otherwise. Residents find the question segment of each lecture to be especially beneficial, as it allows them to gain further information from subject matter experts on specific cases from their own practice. Attendings and residents find the recorded archives helpful as well and report that they refer to it often for review.

## Discussion

The main goal of these grand rounds is to provide educational training opportunities for Haitian orthopedic surgeons to further help their career development. In conjunction with the leadership at major state hospitals in Haiti, ORSI has organized a comprehensive year-round orthopedic surgical lecture series that addresses this goal to provide Haitian residents with a strong foundation in the basics of orthopedics. It has been possible to address a broad range of topics that are pertinent to orthopedics in Haiti, including trauma, pediatrics, orthopedic oncology, and even metabolic bone disease. By training these surgeons further, the series is generating knowledgeable teachers, thus contributing to the education of further generations.

It has been important to follow feedback regarding the content of each presentation. Initially, lectures focused on complex technology and techniques from the US, but Haitian residents reported that these presentations weren’t pertinent to their practice. By following feedback, the program has evolved over the years to successfully address the case mix and infrastructural capacity more appropriately.

While giving a lecture solely on modern orthopedic technologies that are not available in Haiti is inappropriate, dedicating a small part of the lecture series to such topics is still valuable in demonstrating to the orthopedic community what is possible with proper infrastructural support. Resource limited settings should aspire, with proper support, to evolve their technologies to meet standards for modern medicine. Within this lecture series, the future goal is that the orthopedic surgeon who lectures on a particular topic and technology can ultimately serve as a champion and philanthropist to bring this technology to Haiti.

To date, no formal data has been collected to assess the impact of these lectures on the clinical skills of Haitian residents, however feedback gathered after lecture sessions and on a quarterly basis through survey feedback has been overwhelmingly positive from both residents and attendings. Residents report that the lectures provide a good foundation for their daily practice. The question and discussion section at the end of each session has continuously received praise from residents. They look forward to these sessions to ask about current cases and look to their US preceptors for advice. In the future, competency measures will be added to determine the efficacy of the lectures being presented, in addition to measuring resident and attending satisfaction with the program.

A secondary goal of this program is to build relationships between Haitian and American orthopedic surgeons. By building relationships, a further understanding of the needs of Haitians can be learned so that the program can grow and continue to provide more help to colleagues there. In the past, many of the American orthopedic residents that have visited Haiti to work along their Haitian counterparts have been inspired through those experiences to continue to volunteer services in resource limited settings. Programs such as these not only provide for medical knowledge, but also offer the distinct advantage of bidirectional learning and added cultural competency [13]. Furthermore, studies have shown that participation in international surgical missions, especially as residents, make doctors significantly more likely to return for additional trips or to provide further support in the future [14].

These educational programs carry even more significance currently, as didactic teams have been unable to travel to Haiti due to political insecurity and instability. Barriers like this exist around the world as well. Expanding this program to other clinical teaching programs can allow for expanded educational services to orthopedists beyond Haiti; residents from Honduras, Benin, Burkina Faso, and Ethiopia have recently joined conferences as well as the program continues to grow. In the field of orthopedics, case mixes tend to be similar in nature in resource-limited settings, therefore this lecture series has great application globally in such locations.

Furthermore, similar challenges in travel and access to education exist throughout the world in many areas of medicine. While this program has focused on orthopedic surgery, many specialties could create similar programs to benefit countries in need. As noted previously, orthopedic clinical needs and capabilities are similar in resource-limited settings, however this applies to most areas of medicine as well. Programs like this can be designed and widely broadcast to resource impoverished areas of the world.

## Conclusion

“If you give a man a fish, you feed him for a day. If you teach a man to fish, you feed him for a lifetime.” Providing quality orthopedic educational resources to Haitian residents and attendings is an essential supplemental tool that strengthens their training pipeline and is an investment in their ability to be self-sustainable [15,16]. By utilizing virtual modalities to broadcast and archive videos, even more opportunities are available to strengthen medical curriculums. Virtual modalities have the ability to make expansion to other locations seamless, as many programs from across the world may join sessions or watch recordings.

Further measurable outcomes-based data may be required to demonstrate the benefit of educational grand rounds series. However, based on feedback obtained thus far it is clear that this model can be used as a guide to develop similar educational programs in other medically underserved populations to improve access to education throughout the world.
